# Commentary: Efficacy and safety of the innovative monoclonal antibodies in adults with generalized myasthenia gravis: a Bayesian network analysis

**DOI:** 10.3389/fimmu.2024.1403802

**Published:** 2024-05-17

**Authors:** Ina Zhang, Jeroen P. Jansen, Benjamin J. Yungher, Adrian Kielhorn, Karen S. Yee

**Affiliations:** ^1^ Evidence Synthesis and Decision Modeling, PRECISIONheor, Oakland, CA, United States; ^2^ Alexion, AstraZeneca Rare Disease, Boston, MA, United States

**Keywords:** generalized myasthenia gravis, monoclonal antibody, FcRn inhibitor, complement inhibitor, B-cell targeting therapy, network meta-analysis

## Introduction

We read with interest the recent article by Chen et al., 2023 reporting results of their network meta-analysis (NMA) comparing interventions for generalized Myasthenia Gravis (gMG) (1). NMA is an established technique to estimate relative treatment effects among competing interventions based on the available randomized controlled trials (RCTs). NMA findings are relevant for healthcare decision-making when the compared interventions are appropriate for the same target population of interest (2, 3). In essence, we consider the comparisons made in an NMA as relevant if it would make sense to compare all the NMA interventions simultaneously in a new head-to-head RCT (assuming this would be ethically defendable). The credibility of an NMA hinges upon whether studies included in the analysis are representative of the target population of interest and do not differ systematically in their distributions of treatment effect-modifiers. Treatment effect-modifiers are study design characteristics, patient characteristics, or contextual factors that impact the observed treatment effect for a particular study intervention (4). With this in mind, we would like to highlight key aspects of the NMA by Chen et al. that limits their findings to inform decision making and difficult to apply in routine clinical care.

## Discussion

Chen et al. did not explicitly define the target population of interest for their NMA. This is crucial from a relevance perspective because it defines which interventions are relevant to compare and to whom the results are applicable. The NMA aims to compare the efficacy of monoclonal antibodies across multiple treatment classes including FcRn inhibitors, complement inhibitors, and B-cell targeting therapies for gMG. Unfortunately, Chen et al. did not specify whether the target population of interest for their NMA are anti-acetylcholine receptor antibody-seropositive (AChR+) patients, muscle-specific kinase antibody-seropositive (MuSK+) patients, or a mixed population. According to the Food and Drug Administration, eculizumab, ravulizumab, zilucoplan, efgartigimod are indicated for patients with gMG who are AChR+, whereas rozanolixizumab is indicated for patients who are AChR+ or MuSK+ (5–9). Complement inhibitors (eculizumab, ravulizumab, zilucoplan) are not considered suitable for MUSK+ patients (10). Comparing eculizumab, ravulizumab, zilucoplan, efgartigimod, and rozanolixizumab in a single NMA, as done by Chen et al., implies that the actual target population of their interest is the AChR+ gMG population. Without an explicit statement of the target population in the study objective, readers may be confused as to which population the findings are applicable thereby undermining clinical relevance. Worse, readers may mistakenly conclude that comparisons and findings of the NMA are reflective of any gMG population.

A clearly defined target population is also important to judge the credibility of the NMA: Are the trials included appropriate and provide relevant evidence for the target population of interest? The trials for eculizumab (REGAIN), ravulizumab (CHAMPION-MG), and zilucoplan (RAISE) did not include MuSK+ patients, which ensures that the observed trial-specific results are representative of the presumed AChR+ target population of interest and can be included as such in the NMA (11–13). Positive serology for MuSK was not an exclusion criterion in the MycarinG trial evaluating rozanolixizumab or the ADAPT trial evaluating efgartigimod (14, 15). Although the proportion of MuSK+ patients in MycarinG or ADAPT is relatively small, this raises the question whether the trial specific treatment effects of MycarinG or ADAPT as used in the NMA is reflective of an AChR+ target population. This same question applies to other trials for which the study population was not restricted to AChR+ patients and for which there is no access to AChR+ subgroup data (16–19). If this is not the case, the NMA results are “externally biased” relative to the AChR+ target population of interest.

Regarding the risk of a biased NMA due to differences in effect-modifiers between studies, the authors mentioned differences in patient characteristics at baseline of the trials but did not discuss which factors are likely to impact study-specific relative treatment effects. In our opinion, potential treatment-effect modifiers worth exploring relate to disease severity and include baseline MG-ADL, QMG, and MG-QoL scores. The REGAIN, CHAMPION-MG, and RAISE trials enrolled patients with MG-ADL score ≥6, whereas ADAPT and MycarinG enrolled patients with lower severity (MG-ADL score ≥5 and ≥3, respectively). Ignoring these differences in baseline MG-ADL among-studies implies that the relative treatment effects of all monoclonal antibodies are the same for a population with higher MG-ADL as for a population with lower MG-ADL scores at the start of therapy. At present, it is unclear whether this is a reasonable model assumption. If one would have access to individual patient-level data for some or all included trials, one could attempt to adjust for this potential bias in the NMA.

Another limitation not discussed by Chen et al. concerns the differences in timing of outcome assessments across the included RCTs in which the endpoints varied between 43 days (MycarinG) to 52 weeks (BeatMG). Treatment effects observed in studies with a short duration may represent a biased estimate for long-term benefits if those effects do not persist over time. Similarly, treatment effects observed at longer follow-up may not be representative of early treatment effects. Two indirect treatment comparison studies comparing ADAPT with REGAIN and ADAPT with CHAMPION-MG have illustrated how treatment effects at different timepoints can vary (20, 21). As such, timing of outcome assessment may be an effect-modifier, and comparing treatment effects from different time points among trials can compromise the credibility of the NMA as well (22).

## Conclusion

NMA is a powerful evidence synthesis method. However, it is essential to clearly define the target population of interest such that compared interventions in the NMA are considered appropriate for this target population to provide valuable information on how competing interventions “stack up” against one another and help inform clinical decision-making. In addition, detailed considerations of potential differences in treatment effect modifiers among trials included in the NMA should be provided to ensure the interested reader can judge the credibility of NMA findings. The importance of addressing relevance and credibility concerns in an NMA to ensure clinical meaningfulness is evident from the contrasting conclusions in other NMAs evaluating newer therapies for gMG (23, 24).

## Author contributions

IZ: Writing – original draft, Writing – review & editing. JJ: Writing – original draft, Writing – review & editing. BY: Writing – review & editing. AK: Writing – review & editing. KY: Writing – review & editing.
